# Vaccine effectiveness of heterologous CoronaVac plus BNT162b2 in Brazil

**DOI:** 10.1038/s41591-022-01701-w

**Published:** 2022-02-09

**Authors:** Thiago Cerqueira-Silva, Srinivasa Vittal Katikireddi, Vinicius de Araujo Oliveira, Renzo Flores-Ortiz, Juracy Bertoldo Júnior, Enny S. Paixão, Chris Robertson, Gerson O. Penna, Guilherme L. Werneck, Maurício L. Barreto, Neil Pearce, Aziz Sheikh, Manoel Barral-Netto, Viviane S. Boaventura

**Affiliations:** 1 LIB and LEITV Laboratories, Instituto Gonçalo Moniz, Salvador, Brazil; 2 Universidade Federal de Bahia (UFBA), Salvador, Brazil; 3 MRC/CSO Social and Public Health Sciences Unit, University of Glasgow, Glasgow, UK; 4 Public Health Scotland, Glasgow, UK; 5 Center of Data and Knowledge Integration for Health (CIDACS), Instituto Gonçalo Moniz, Salvador, Brazil; 6 London School of Hygiene and Tropical Medicine, London, UK; 7 Department of Mathematics and Statistics, University of Strathclyde, Glasgow, UK; 8 Núcleo de Medicina Tropical, Universidade de Brasília, Escola Fiocruz de Governo, Fiocruz, Brazil; 9 Universidade do Estado do Rio de Janeiro, Rio de Janeiro, Brazil; 10 Usher Institute, University of Edinburgh, Edinburgh, UK

**Keywords:** Epidemiology, Outcomes research

## Abstract

There is considerable interest in the waning of effectiveness of coronavirus disease 2019 (COVID-19) vaccines and vaccine effectiveness (VE) of booster doses. Using linked national Brazilian databases, we undertook a test-negative design study involving almost 14 million people (~16 million tests) to estimate VE of CoronaVac over time and VE of BNT162b2 booster vaccination against RT–PCR-confirmed severe acute respiratory syndrome coronavirus 2 (SARS-CoV-2) infection and severe COVID-19 outcomes (hospitalization or death). Compared with unvaccinated individuals, CoronaVac VE at 14–30 d after the second dose was 55.0% (95% confidence interval (CI): 54.3–55.7) against confirmed infection and 82.1% (95% CI: 81.4–82.8) against severe outcomes. VE decreased to 34.7% (95% CI: 33.1–36.2) against infection and 72.5% (95% CI: 70.9–74.0) against severe outcomes over 180 d after the second dose. A BNT162b2 booster, 6 months after the second dose of CoronaVac, improved VE against infection to 92.7% (95% CI: 91.0−94.0) and VE against severe outcomes to 97.3% (95% CI: 96.1−98.1) 14–30 d after the booster. Compared with younger age groups, individuals 80 years of age or older had lower protection after the second dose but similar protection after the booster. Our findings support a BNT162b2 booster vaccine dose after two doses of CoronaVac, particularly for the elderly.

## Abstract (web summary)

A test-negative case–control analysis of data from Brazil shows that vaccine effectiveness against SARS-CoV-2 infection and severe COVID-19 outcomes declines after two doses of CoronaVac but increases after a booster dose of the BNT162b2 vaccine.

## Main

Vaccination is an essential strategy to mitigate the effects of the coronavirus disease 2019 (COVID-19) pandemic. Inactivated virus vaccines are among the most widely used worldwide, and they are especially useful for low- and middle-income countries given their less stringent cold chain requirements for preservation and transportation and their lower costs compared to mRNA vaccines. The most commonly used inactivated virus vaccines are CoronaVac, Sinopharm and Bharat Biotech, with more than 4.5 billion doses of these vaccines having been delivered worldwide as of 14 December 2021 (ref. ^1^).

Initial evaluations of VE of inactivated virus vaccines have demonstrated high protection against severe disease, especially in the non-elderly population^2,3^. However, even in younger individuals, VE is lower compared to other vaccine types^3^. There is growing evidence of waning protection against severe acute respiratory syndrome coronavirus 2 (SARS-CoV-2) infection and severe COVID-19 outcomes for mRNA-based vaccines coding for the spike protein^4,5^. Neutralizing antibody responses wane after CoronaVac vaccination^6,7^, but the durability of VE against clinical outcomes is unknown and has important implications for informing decisions about vaccine boosters.

In Brazil, COVID-19 vaccination started on 18 January 2021. The Brazilian COVID-19 vaccination program now includes CoronaVac (Sinovac Biotech), ChAdOx1 (AstraZeneca), Ad26.COV2.S (Janssen) and BNT162b2 (Pfizer-BioNTech) vaccines for primary immunization. All doses administered in Brazil were provided by the Ministry of Health. CoronaVac was the first COVID-19 vaccine to be offered and was the most widely used among individuals 60 years of age and older. By the end of June, most elderly individuals had received two doses of CoronaVac (primary vaccination series)^3^. Six months after completing a primary vaccination series, individuals become eligible for a booster dose, preferentially with BNT162b2 vaccine^8^.

Using Brazilian national data, we evaluated the effectiveness of two doses of CoronaVac against confirmed SARS-CoV-2 infection and severe COVID-19 outcomes (hospitalization and death) from time since vaccination compared to unvaccinated individuals, using a test-negative design (TND) case–control study. We also estimated the effectiveness of the BNT162b2 mRNA vaccine as a booster dose, which has been the most widely used booster vaccine in Brazil. A summary of the main findings, limitations and policy implications of the study is provided in Table 1.

**Table 1:** Policy summary

**Background**	Protection of mRNA-based and viral vector-based vaccines against infection, hospital admission and death due to SARS-CoV-2 declines over time, with these effects being most pronounced among the elderly. It is unclear whether similar patterns of waning are seen for inactivated whole-cell COVID-19 vaccines. In Brazil, CoronaVac has been administered since January 2021, and most older individuals received this vaccine. In September 2021, a booster vaccine dose program began, this being offered to individuals 6 months after completing their primary vaccination schedule. The BNT162b2 mRNA vaccine was primarily used for boosters.
**Main findings and limitations**	Analyzing linked national Brazilian databases, we observed that protection against infection, hospitalization and death fell over time after the primary vaccination schedule with CoronaVac, with particularly marked decreases in older individuals. The decline in VE occurred in the context of Gamma and Delta being the dominant viral variants. A BNT162b2 mRNA booster dose restored VE against infection and severe outcomes in all age groups.
	Some limitations include the short length of follow-up after the booster dose and analyzing only BNT162b2 mRNA boosters. Changes in transmission rates and in the viral variants circulating during the study period might have influenced VE over the time.
**Policy implications**	Our findings provide evidence for using a heterologous booster of the BNT162b2 mRNA vaccine after completing the primary CoronaVac immunization schedule to achieve a sufficient level of protection against infection and severe outcomes. Continuous monitoring of VE will be necessary to evaluate the duration of protection after the booster dose.

## Results

**Fig. 1:**
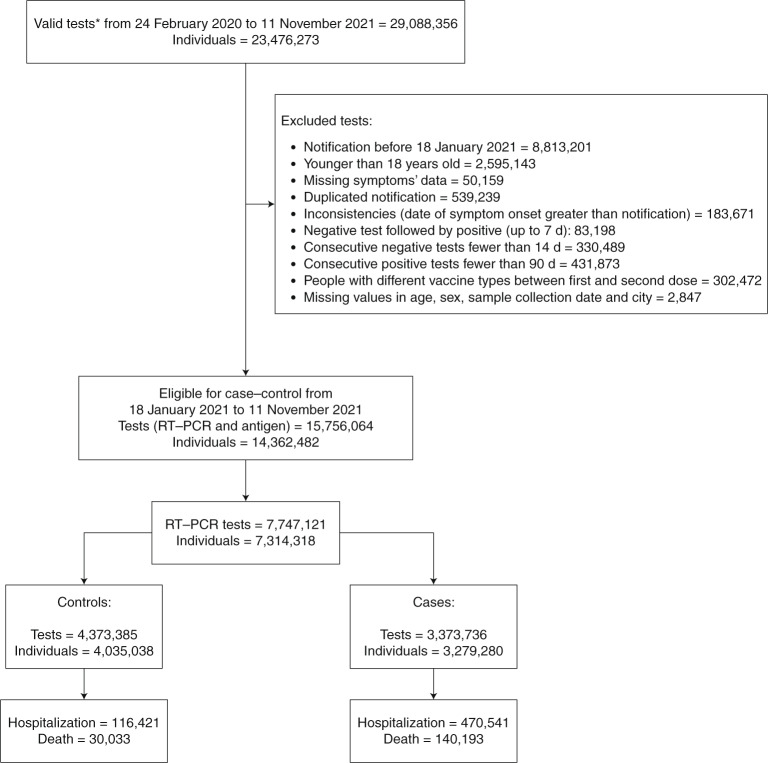
Flowchart of the study population from surveillance databases and selection of cases and controls. *Antigen or RT–PCR—sample collected 10 or fewer days after symptom onset.

**Extended Data Fig. 1:**
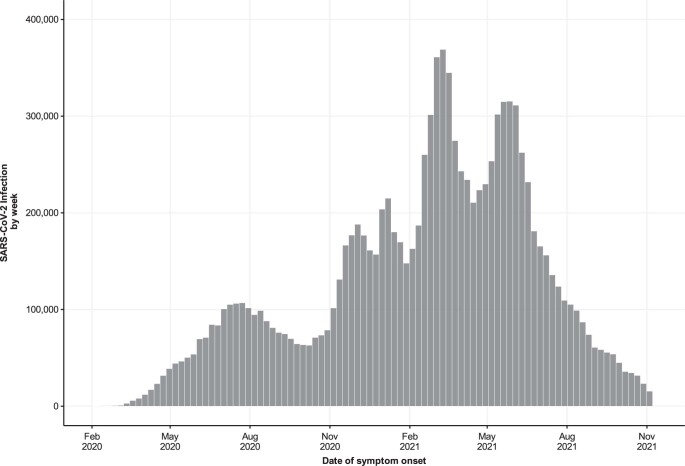
Cases of infection by SARS-CoV-2 from February 2020 to November,11 2021, by week.

**Extended Data Fig. 2:**
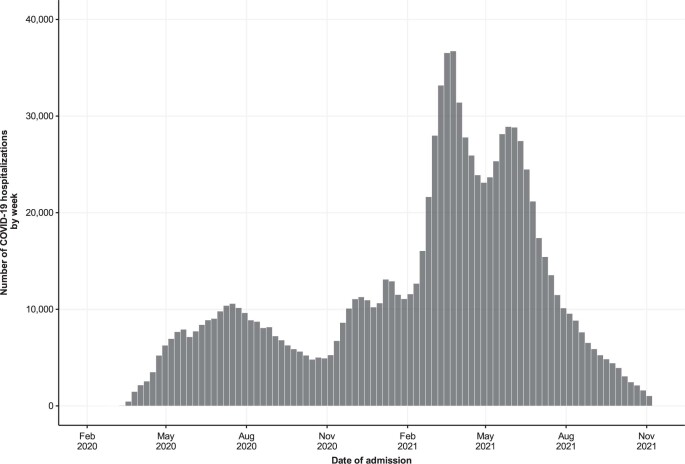
Distribution of hospitalization due to COVID-19 from February 2020 to November 11, 2021, by week.

**Extended Data Fig. 3:**
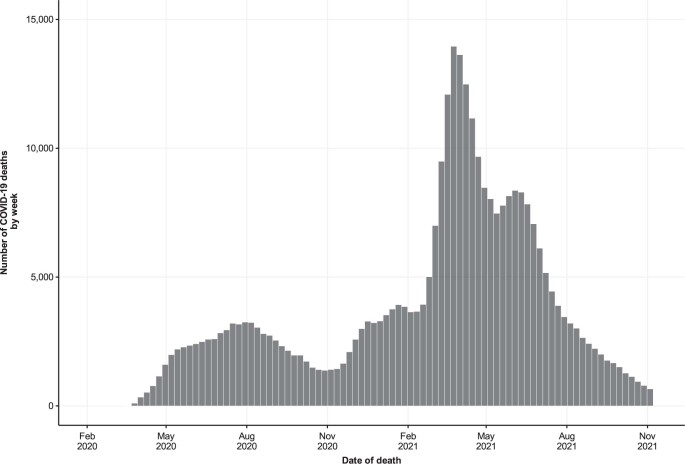
Distribution of deaths due to COVID-19 from February 2020 to November 11, 2021, by week.

**Extended Data Fig. 4:**
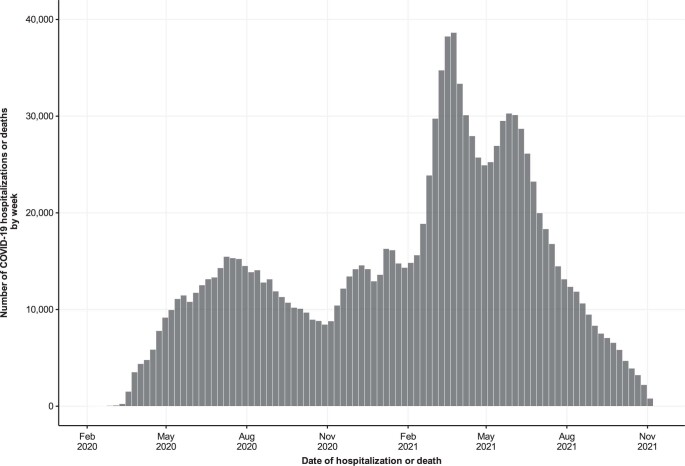
Distribution of severe outcomes due to COVID-19 from February 2020 to November 11, 2021, by week.

From 24 February 2020 to 11 November 2021, 23,476,273 individuals were tested for suspected SARS-CoV-2 infection, with a peak of severe outcomes between February 2021 and April 2021 (Extended Data Figs. 1–4). Among the 13.3 million tests not eligible for this study, 8.8 million were performed before the vaccination campaign in Brazil (18 January 2021). These tests were used only to access the status of the previous infections of the study participants and were not used in the main analysis. Additionally, 2.6 million tests were performed in individuals younger than 18 years of age, an age group not included in the present study. In the study period—18 January 2021 to 11 November 2021—a total of 14,362,482 individuals were considered eligible and were tested either by rapid antigen test or RT–PCR, of whom 7,314,318 individuals (7,747,121 tests) were tested by RT–PCR (Fig. 1).

**Fig. 2:**
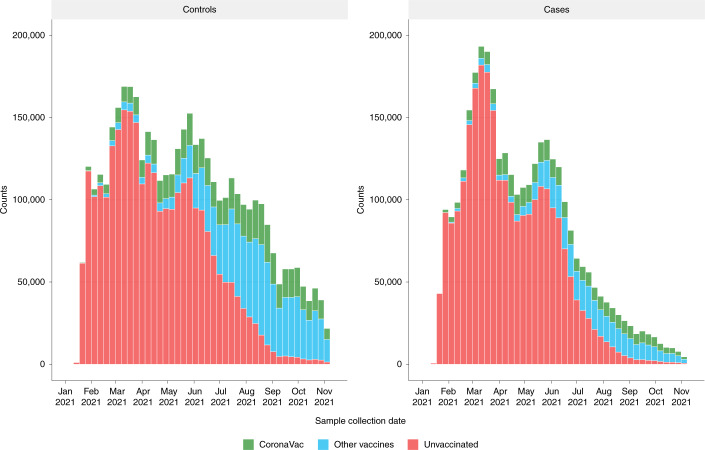
Number of cases and controls, by week, during the study period, stratified by vaccination status (unvaccinated, vaccinated with CoronaVac and vaccinated with other vaccines). Green, individuals with at least one dose of CoronaVac. Blue, individuals with at least one dose of any other vaccine (BNT162b2, ChAdOx1 or Ad26.COV2.S). Red, individuals unvaccinated.

**Extended Data Fig. 5:**
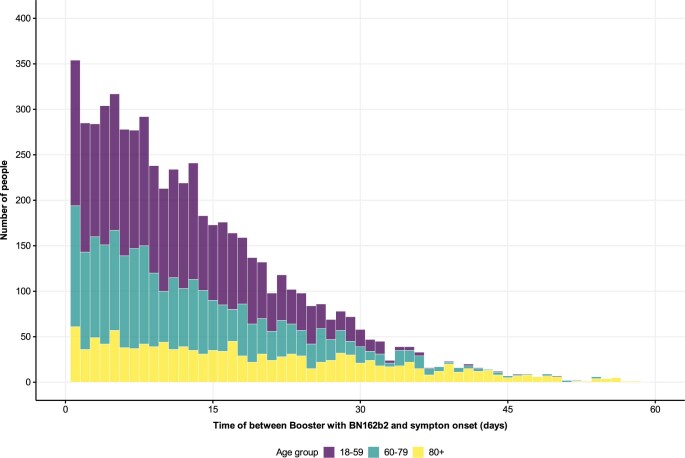
Distribution of time between booster dose and symptom onset, in days, by age group.

**Extended Data Fig. 6:**
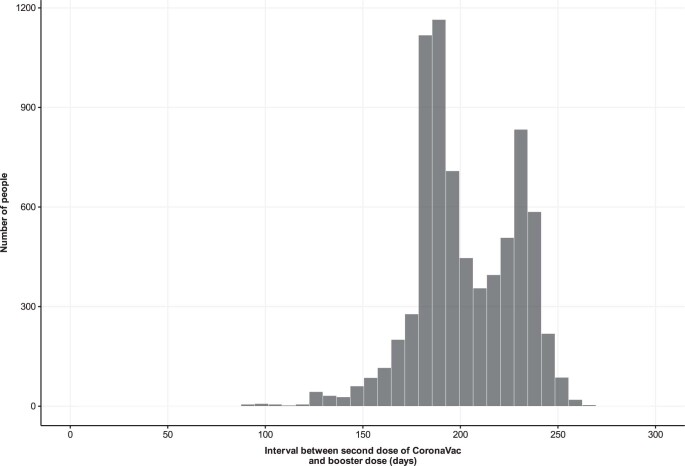
Distribution of time elapsed between second dose and booster dose in the population of study.

Most individuals (71.4%) were unvaccinated at the time of testing, but there was a sharp decrease in the number of tests per week after June 2021 and a gradual transition to more vaccinated individuals being tested, corresponding to the increase in the cumulative uptake of vaccination in Brazil (Fig. 2 and Supplementary Fig. 1). A total of 913,052 individuals were vaccinated with CoronaVac, of whom 7,863 received a booster dose of BNT162b2. Most of these individuals (93.4%) were tested within 30 d after the booster dose (Table 2, Extended Data Tables 1 and 2 and Extended Data Figs. 5 and 6).

**Table 2:** Clinical and sociodemographic characteristics of individuals included in a TND analysis by SARS-CoV-2 RT–PCR positivity

Characteristic	Controls, *n* = 4,373,385	Cases, *n* = 3,373,736	Overall, *n* = 7,747,121
Individuals	4,035,038 (92%)	3,279,280 (97%)	7,314,318 (94%)
Age, years (median IQR)	37 (28, 49)	41 (31, 54)	39 (29, 51)
Sex, female	2,457,036 (56.2%)	1,744,427 (51.7%)	4,201,463 (54.2%)
Race			
White	1,895,837 (43.3%)	1,311,471 (38.9%)	3,207,308 (41.4%)
Black	206,689 (4.7%)	145,220 (4.3%)	351,909 (4.5%)
Asian	74,183 (1.7%)	52,994 (1.6%)	127,177 (1.6%)
Mixed	1,369,960 (31.3%)	1,159,865 (34.4%)	2,529,825 (32.7%)
Indigenous	5,425 (0.1%)	2,698 (0.1%)	8,123 (0.1%)
(Missing)	821,291 (18.8%)	701,488 (20.8%)	1,522,779 (19.7%)
Age group			
18–59	3,838,544 (87.8%)	2,820,645 (83.6%)	6,659,189 (86.0%)
60–79	453,589 (10.4%)	473,094 (14.0%)	926,683 (12.0%)
≥80	81,252 (1.9%)	79,997 (2.4%)	161,249 (2.1%)
Region of residence			
Central West	835,224 (19.1%)	466,542 (13.8%)	1,301,766 (16.8%)
North	208,015 (4.8%)	155,470 (4.6%)	363,485 (4.7%)
Northeast	784,288 (17.9%)	718,474 (21.3%)	1,502,762 (19.4%)
South	330,917 (7.6%)	325,544 (9.6%)	656,461 (8.5%)
Southeast	2,214,941 (50.6%)	1,707,706 (50.6%)	3,922,647 (50.6%)
Pregnancy	47,568 (1.1%)	18,990 (0.6%)	66,558 (0.9%)
Postpartum period	3,429 (0.1%)	2,136 (0.1%)	5,565 (0.1%)
Number of comorbidities			
0	3,921,752 (89.7%)	2,875,746 (85.2%)	6,797,498 (87.7%)
1	346,883 (7.9%)	351,777 (10.4%)	698,660 (9.0%)
≥2	104,750 (2.4%)	146,213 (4.3%)	250,963 (3.2%)
Previous confirmed infection	233,810 (5.3%)	39,593 (1.2%)	273,403 (3.5%)
Vaccination status			
Unvaccinated	2,868,434 (65.6%)	2,661,562 (78.9%)	5,529,996 (71.4%)
CoronaVac	590,266 (13.5%)	322,786 (9.6%)	913,052 (11.8%)
Other vaccines	914,685 (20.9%)	389,388 (11.5%)	1,304,073 (16.8%)
Hospitalization^a^	116,421 (2.7%)	470,541 (14%)	586,962 (7.6%)
Death^a^	30,033 (0.7%)	140,193 (4.2%)	170,226 (2.2%)
Hospitalization or death^a^	119,125 (2.7%)	477,751 (14.2%)	596,876 (7.7%)

IQR, interquartile range; *n* (%).

^a^Related to COVID-19 (for cases) and related to other acute respiratory illness (control).

### Main analyses

Compared to unvaccinated individuals, VE against infection and severe outcomes progressively decreased with time from the second CoronaVac dose and increased after the BNT162b2 booster dose (Tables 3 and 4). Waning protection was more marked for infection than for severe COVID-19 outcomes. Between 14–30 d and more than 180 d after the second dose, VE against infection decreased from 55.0% (95% confidence interval (CI): 54.3–55.7) to 34.7% (95% CI: 33.1–36.2). There was an increase in VE 7–13 d after BNT162b2 booster vaccination (80.2%, 95% CI: 77.0–82.9), reaching a peak at 14–30 d (92.7%, 95% CI: 91.0–94.0). A decrease in VE against infection was observed 30 d after the booster dose (82.6%, 95% CI: 76.9–86.9); however, this group represents only 6.6% of individuals who received a booster dose in our sample (Table 3 and Extended Data Table 1).

**Table 3:** Effectiveness of CoronaVac vaccine against confirmed SARS-CoV-2 infection, by length of time (in days) since two-dose vaccination or BNT162b2 booster dose, stratified by age group

Period after vaccine (days)	Overall	18–59	60–79	≥80
**Second dose**				
0–13	37.9% (36.9–38.8)	43.5% (42.4–44.7)	32.2% (30.1–34.2)	28.3% (23.4–32.9)
14–30	55.0% (54.3–55.7)	56.5% (55.6–57.5)	55.1% (53.7–56.5)	50.3% (46.8–53.6)
31–60	51.7% (51.1–52.4)	52.9% (52.1–53.8)	51.1% (49.7–52.4)	47.0% (43.7–50.1)
61–90	47.6% (46.8–48.3)	48.9% (47.9–49.9)	45.3% (43.6–46.9)	41.0% (37.3–44.4)
91–120	46.1% (45.3–46.9)	52.3% (51.3–53.2)	39.8% (37.8–41.8)	31.8% (27.3–36.1)
121–150	41.8% (40.8–42.8)	50.6% (49.3–51.9)	36.3% (33.8–38.7)	22.1% (16.5–27.3)
151–180	38.0% (36.7–39.3)	44.0% (42.3–45.6)	35.3% (32.2–38.2)	15.1% (8.3–21.5)
>180	34.7 % (33.1–36.3)	34.1% (32.2–35.9)	34.5% (29.9–38.7)	10.1% (1.1–18.3)
**Booster (BNT162b2)**				
0–6	39.6% (33.8–44.8)	40.3% (31.6–47.8)	35.7% (25.2–44.8)	11.5% (−12.4–30.3)
7–13	80.2% (77.0–82.9)	84.6% (80.2–88.0)	75.9% (69.6–80.8)	59.6% (44.9–70.4)
14–30	92.7% (91.0–94.0)	93.5% (90.7–95.5)	93.4% (90.3–95.5)	82.0% (75.0–87.0)
>30	82.6% (76.9–86.9)	61.8% (27.2–79.9)	81.2% (67.6–89.1)	66.4% (49.6–77.5)

**Table 4:** Effectiveness of CoronaVac vaccine against COVID-19 hospitalization or death, by length of time (in days) since two-dose vaccination or BNT162b2 booster dose, stratified by age group

Period after vaccine (days)	Overall	18–59	60–79	≥80
**Second dose**				
0–13	65.5% (64.2–66.6)	79.6% (77.6–81.4)	64.5% (62.8–66.1)	51.4% (47.3–55.1)
14–30	82.1% (81.4–82.8)	91.4% (90.3–92.4)	81.6% (80.6–82.5)	68.7% (65.9–71.2)
31–60	82.6% (82.1–83.2)	89.9% (88.9–90.9)	81.4% (80.6–82.2)	66.5% (64.0–68.9)
61–90	80.5% (79.8–81.0)	87.2% (86.0–88.3)	77.6% (76.6–78.6)	63.2% (60.4–65.8)
91-120	78.9% (78.3–79.6)	89.0% (87.8–90.0)	75.5% (74.3–76.7)	58.0% (54.7–61.1)
121–150	77.0% (76.1–77.8)	86.7% (85.2–88.0)	74.9% (73.5–76.3)	52.1% (48.0–55.8)
151–180	75.0% (73.9–76.0)	81.9% (79.8–83.8)	74.7% (72.9–76.4)	47.9% (42.9–52.4)
>180	72.6% (71.0–74.2)	74.8% (72.1–77.2)	72.6% (69.5–75.3)	41.4% (34.5–47.5)
**Booster (BNT162b2)**				
0–6	80.6% (76.4–84.0)	89.1% (76.6–94.9)	79.6% (73.5–84.2)	48.8% (31.3–61.9)
7–13	91.4% (88.5–93.5)	95.8% (82.9–99.0)	88.3% (83.1–91.8)	78.0% (67.1–85.3)
14–30	97.3% (96.1–98.1)	97.9% (85.0–99.7)	97.1% (94.7–98.5)	89.5% (83.9–93.1)
>30	96.8% (94.1–98.3)	100% (*)	92.0% (79.6–96.9)	89.3% (78.6–94.7)

*The CI could not be estimated owing to zero/few events in the group.

Estimated VE for severe disease also decreased from 82.1% (95% CI: 81.4–82.8) at 14–30 d to 72.5% (95% CI: 70.9–74.0) more than 180 d after the second CoronaVac dose (Table 3). VE then increased gradually after the BNT162b2 booster dose to 80.6% (95% CI: 76.4–84.0) at 0–6 d, 97.3% (95% CI: 96.1–98.1) at 14–30 d and 96.8% (95% CI: 94.1–98.3) 30 d after booster vaccination (Table 4).

### Subgroup analyses by age and outcome

We also conducted an analysis by age groups of VE after CoronaVac and VE after the BNT162b2 booster. The comparison for VE estimates was unvaccinated individuals. In individuals 80 years of age or older, the pattern of waning was more accentuated. The VE against infection fell from 50.3% (95% CI: 46.8–53.6) at 14–30 d to 10.1% (95% CI: 1.1–18.3) at more than 180 d after the second dose of CoronaVac. After the booster dose of BNT162b2, VE against infection reached 82.0% (95% CI: 75.0–87.0) at 14–30 d and 66.4% (95% CI: 49.6–77.5) at more than 30 d after the booster dose. Protection against severe disease fell from 68.7% (95% CI: 65.9–71.2) at 14–30 d to 41.0% (95% CI: 34.1–47.3) at more than 180 d after the second dose with CoronaVac (Table 4). A marked increase in VE was observed at 14–30 d (89.5%, 95% CI: 83.9–93.1) and over 30 d (89.3%, 95% CI: 78.6–94.7) after administration of the booster dose (Table 4). Waning of protection was also observed for individuals aged 18–59 and 60–79 years, although to a lesser extent, with increases in VE after the booster dose (Tables 3 and 4).

The effectiveness against hospitalization after the primary vaccination series with CoronaVac at 14–30 d after the second dose was 82.1% (95% CI: 81.4–82.8) and 72.4% (95% CI: 70.7–73.9) at more than 180 d after the second dose. Fourteen to 30 d after the BNT162b2 booster dose, this increased to 97.2% (95% CI: 96.0–98.0). Similar results were observed for protection against death. VE against death varied from 82.7% (95% CI: 81.7–83.6) at 14–30 d to 74.8% (95% CI: 72.2–77.2) at more than 180 d after the second dose and increased to 98.3% (95% CI: 96.3–99.2) 14–30 d after the BNT162b2 booster dose. Both hospitalizations and deaths had a similar pattern compared to the composite outcome when stratified by age group (Extended Data Tables 3 and 4).

### Sensitivity analyses

Given that there was an increase in the use of rapid antigen tests as a diagnostic tool in Brazil, despite its lower accuracy compared to RT–PCR tests, we performed a sensitivity analysis including both diagnostic tests as inclusion criteria. Similar results were obtained when an antigen detection test was used in addition to RT–PCR to define the clinical outcome (Supplementary Table 2). Using rapid antigen plus RT–PCR tests as diagnostic criteria, VE against SARS-CoV-2 infection between 14 and 30 d after the booster was 97.2% (95% CI: 96.0–98.0), which was similar to VE for the main analysis using only RT–PCR tests in this period. We observed that VE against SARS-CoV-2 infection more than 30 d after the booster was different when comparing rapid antigen plus RT–PCR tests (96.7%, 95% CI: 93.9–98.2) to only RT–PCR (82.6%, 95% CI: 76.9–86.9). This difference when compared to the estimates using RT–PCR alone is likely due to the increase in the sample size (Supplementary Tables 1 and 2).

To untwine the roles of the recent update of booster doses and circulation of the Delta variant, we assessed the additional protection offered by the booster dose compared to individuals who received only two doses of CoronaVac in the period of predominant circulation of the Delta variant (August–November 2021). We evaluated VE at peak response after the booster dose (14–30 d) compared to VE in those who had completed the two-dose CoronaVac immunization regimen more than 180 d beforehand. At 14–30 d after the booster dose, VE against infection was 88.8% (95% CI: 86.3–90.8), and VE against severe outcome was 90.1% (95% CI: 85.7–93.1), relative to those two-dose CoronaVac vaccinated over 180 d ago (Supplementary Tables 4 and 5).

## Discussion

The overall VE of CoronaVac against confirmed SARS-CoV-2 infection and COVID-19 hospitalization or death waned over time. Protection rebounded, reaching values higher than observed for the two-dose regimen, after a booster dose with BNT162b2. To our knowledge, this is the first study to demonstrate the VE of heterologous prime booster vaccination using an inactivated vaccine and an mRNA vaccine booster.

Our findings on the waning protection of CoronaVac and the effect of a heterologous prime boost dose are consistent with studies evaluating long-term immune responses after inactivated virus vaccines^7,9–11^. Vaccine-induced antibodies declined with time after CoronaVac vaccination^9^ and increased when a booster dose of BNT162b complemented the two-dose schedule of inactivated vaccine in humans and in an animal model^11,12^. These findings support the use of an mRNA vaccine booster dose in individuals immunized with two doses of CoronaVac. Other issues, such as the inequity of access to the vaccine and the emergence of new variants, should also be considered in addition to VE when including booster doses in vaccine schedules.

The waning of VE occurred in all age groups but was most evident in the elderly. Similar findings, albeit of different magnitudes, have also been found for BNT162b2 and ChAdOx1 vaccines, and lower VE for the elderly has been previously reported for CoronaVac^3,13–15^. Immunologic protection markers, such as neutralizing antibodies, were also less frequently detected in older versus younger individuals after vaccination with CoronaVac^16^. As CoronaVac was the first vaccine administered in Brazil, with high uptake in the elderly, there is an over-representation of elderly individuals with longer follow-up. In the present report, although individuals 80 years and older had less than 50% protection against severe COVID-19 5 months after the second CoronaVac dose, VE was over 70% for vaccinees younger than 80 years of age 6 months after the second dose. Considering that older adults are at increased risk of severe outcomes, these results reinforce the necessity of closely monitoring VE for this population.

A strength of our study was the use of large-scale, high-quality, routinely collected real-world data from Brazil. The study used a TND, a design that minimizes bias related to access to healthcare, the occurrence of symptoms and health-seeking behaviors. In Brazil, no specific recommendation was made against testing individuals who were vaccinated. Additionally, similar results were obtained in sensitivity analyses using both RT–PCR and antigen tests, demonstrating the robustness of our findings.

This study has several limitations. First, there was a decrease in transmission rates over time in Brazil, but we adjusted for temporal trends in the analyses. Second, it is possible that changes in viral variants might confound our assessment of VE over time. A stepwise increase in the frequency of Gamma and Delta variants of concern was observed in January–July 2021 and in August–October 2021, respectively, which are included in our study period. An additional effect of the Delta variant in time-dependent waning immunity has been demonstrated after vaccination with two doses of CoronaVac^17^. Third, it is difficult to isolate the performance of one vaccine in a scenario with high uptake of three other vaccines.

In conclusion, we have shown that the VE of the two-dose regimen of CoronaVac against both SARS-CoV-2 infection and COVID-19-related severe outcomes waned for all age groups, particularly in the elderly, and protection increased after a BNT162b2 mRNA booster dose. Our findings provide supportive evidence for a marked increase in protection against both infection and severe outcomes after using a heterologous booster of the BNT162b2 mRNA vaccine in addition to the regular immunization schedule of CoronaVac, especially for older people. A longer follow-up period is necessary to understand how long this level of protection lasts.

## Methods

### Study design and data sources

The TND is a type of case–control study that uses population test results, with the positive tests being the cases and the negative tests being the controls. It is ideally suited to situations where not everyone in a population is being tested, because the factors that influence being tested (health-seeking behavior, access to healthcare, availability of testing, etc.) will apply to both those who test positive and those who test negative^18^. We conducted a TND case–control study to assess VE of the two-dose schedule of CoronaVac over time and the booster dose of BNT162b2 on RT–PCR-confirmed SARS-CoV-2 infection and severe COVID-19 outcomes (hospitalization or death) among adults. From January to November 2021, Gamma and Delta variants circulated in Brazil (Supplementary Fig. 2). The Brazilian Ministry of Health recommends 28 d between the first and second doses of CoronaVac. In the second semester of 2021, a booster dose using BNT162b2, ChAdOx1, Ad26.COV2.S or CoronaVac was recommended 6 months after the second CoronaVac dose^15^, although approximately 92% received BNT162b2 (Supplementary Fig. 3).

We analyzed a deterministically linked dataset comprised of the Programa Nacional de Imunizações, which holds records of all vaccines administered in Brazil (BNT162b2, ChAdOx1, Ad26.COV2.S or CoronaVac); the e-SUS Notifica, which contains records of suspected and confirmed COVID-19 in outpatient clinics; and the Sistema de Informação da Vigilância Epidemiológica da Gripe, which holds records of all COVID-19 hospitalizations and deaths. All data were pseudo-anonymized, with a common unique identifier provided by the Brazilian Ministry of Health (Supplementary Fig. 4). The research protocol was approved by the Brazilian National Commission in Research Ethics (CONEP) (approval no. 4.921.308). Our statistical analysis plan is available at https://vigivac.fiocruz.br.

All individuals aged 18 years or older who reported COVID-19-like symptoms and were tested for SARS-CoV-2 between 18 January 2021 and 11 November 2021 were eligible for the study. We excluded: (1) individuals younger than 18 years; (2) individuals who received a different vaccine for the second dose from the first; (3) individuals whose time interval between the first and second doses was fewer than 14 d; (4) tests with missing information of age, sex, city of residence or sample collection date; (5) negative test within 14 d of a previous negative test; (6) negative test followed by a positive test up to 7 d; (7) any test after a positive test up to 90 d; and (8) tests with a symptom onset date greater than notification date (Fig. 1). Cases of confirmed infection were defined as adults with a positive SARS-CoV-2 RT–PCR test and controls with a negative SARS-CoV-2 RT–PCR test, both from a sample collected within 10 d of symptom onset. Cases of COVID-19 hospitalization or death were defined by a positive SARS-CoV-2 test accompanied by hospitalization or death occurring within 28 d of the sample collection date. Controls for the outcome of hospitalization or death were defined based on a negative test.

As a sensitivity analysis, we included SARS-CoV-2 tests based on antigen detection in addition to RT–PCR tests. Antigen test has lower accuracy than RT–PCR test^19^. However, the antigen test has been progressively replacing the RT–PCR test, corresponding to 50.8% of the confirmatory tests for SARS-CoV-2 in the sample population of this study.

### Statistical analysis

The odds ratio (OR) comparing odds of vaccination between cases and controls and its associated 95% CI were derived using generalized additive logistic regression, adjusting for potential confounders identified from previous literature (age, sex, temporal trends, state of residence, previous infection, pregnancy, postpartum period and comorbidities)^20^. The temporal trend was estimated using the time elapsed, in days, between the study start and the date of symptom onset. Temporal trends and age were modeled as cubic regression spline smooth functions. The comorbidities were cardiac disease, diabetes mellitus, obesity, immunosuppression and chronic kidney disease (categorized in the model as none, one and at least two). VE was estimated as 1-OR and expressed as a percentage. Vaccination status, according to the status at the time of RT–PCR test collection, was classified as unvaccinated and grouped in periods (days) after each dose: first dose (0–6, 7–13 and ≥14), second dose (0–13, 14–30, 31–60, 61–90, 91–120, 121–150, 151–180 and >180) and booster dose (0–6, 7–13, 14–30 and >30). Analyses were also performed stratified by age groups (18–59, 60–79 and ≥80 years). As sensitivity analysis, we also compared individuals with booster dose against individuals with second dose over 180 d. All data processing and analyses were performed in R (version 4.1.1)^21^, using the following packages: tidyverse^22^ and mgcv^23^.

### Reporting Summary

Further information on research design is available in the Nature Research Reporting Summary linked to this article.

## Online content

Any methods, additional references, Nature Research reporting summaries, source data, extended data, supplementary information, acknowledgements, peer review information; details of author contributions and competing interests; and statements of data and code availability are available at 10.1038/s41591-022-01701-w.

### Supplementary information

Supplementary Information Supplementary Tables 1–5 and Figs. 1–4.

Reporting Summary

## Figures and Tables

**Extended Data Table 1:**
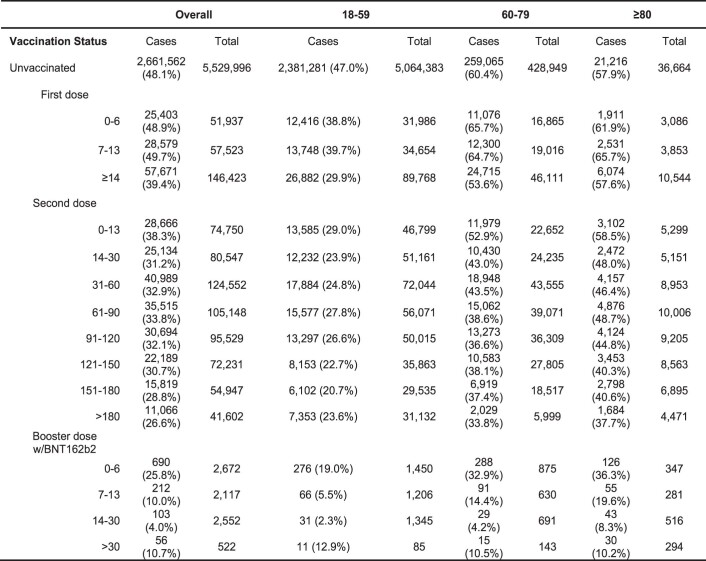
Number of SARS-CoV-2 infection and total of tests of individuals categorized as unvaccinated and vaccinated with CoronaVac

**Extended Data Table 2:**
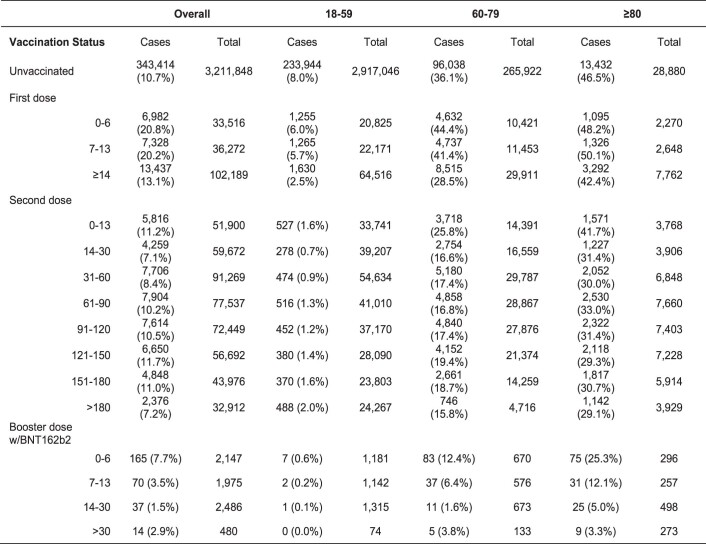
Number of COVID-19 hospitalization or death and total of individuals in unvaccinated and CoronaVac vaccinees

**Extended Data Table 3:**
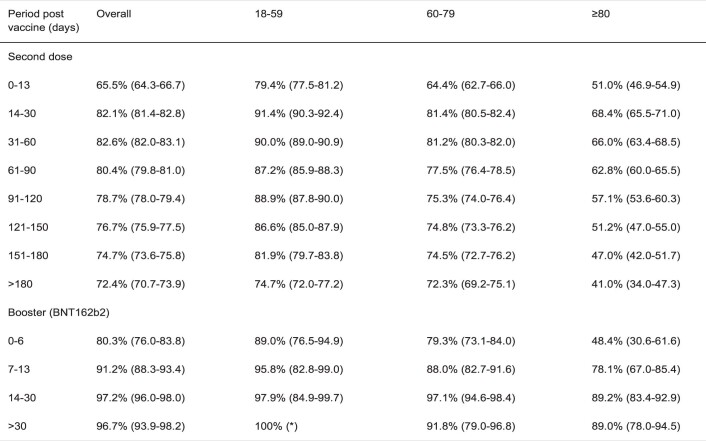
Vaccine effectiveness against hospitalisation due to COVID-19 using RT-PCR, by length of time (in days) since two-dose vaccination or BNT162b2 booster dose

The confidence interval could not be estimated due to zero/few events in the group

**Extended Data Table 4:**
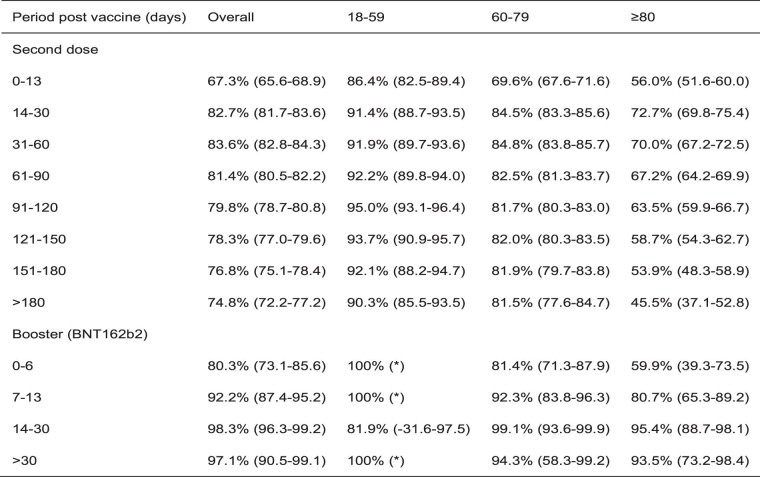
Vaccine effectiveness against death due to COVID-19 using RT-PCR, by length of time (in days) since two-dose vaccination or BNT162b2 booster dose

The confidence interval could not be estimated due to zero/few events in the group

## Data Availability

One of the study coordinators (M.B.-N.) signed a term of responsibility on using each database made available by the Ministry of Health (MoH). Each member of the research team signed a term of confidentiality before accessing the data. Data were manipulated in a secure computing environment, ensuring protection against data leakage. The Brazilian National Commission in Research Ethics approved the research protocol (CONEP approval no. 4.921.308). Our agreement with the MoH for accessing the databases patently denies authorization of access to a third party. Any information for assessing the databases must be addressed to the Brazilian MoH at https://datasus.saude.gov.br/, and requests can be addressed to datasus@saude.gov.br. In this study, we used anonymized secondary data following the Brazilian Personal Data Protection General Law, but it is vulnerable to re-identification by third parties as they contain dates of relevant health events regarding the same person. To protect the research participants’ privacy, the approved Research Protocol (CONEP approval no. 4.921.308) authorizes the dissemination only of aggregated data, such as the data presented here.

## Code availability

All code used in this study is publicly available at https://vigivac.fiocruz.br/category/publicacoes.html.
