# Supplementary material for: Vaccine effectiveness of heterologous CoronaVac plus BNT162b2 in Brazil
Source: Nat Med. 2022 Feb 9;28(4):838–43. doi: 10.1038/s41591-022-01701-w (PMC9018414; doi:10.1038/s41591-022-01701-w)

nature portfolio

https://doi.org/10.1038/s41591-022-01701-w

Supplementary information

Vaccine effectiveness of heterologous CoronaVac plus BNT162b2 in Brazil

In the format provided by the authors and unedited

Supplementary Tables and Figures

Supplementary table 1. Clinical and sociodemographic characteristics of individuals included in the sensitivity analysis, by SARS-CoV-2 RT-PCR or Antigen test positivity. Vaccination status for CoronaVac is categorized by days since receiving a CoronaVac dose and a booster dose of BNT162b2. 2

Supplementary table 2- Vaccine effectiveness against SARS-CoV-2 RT-PCR confirmed symptomatic infection and hospitalization/death using RT-PCR plus antigen detection tests for the diagnosis of COVID-19, by time (days) since second dose of CoronaVac or after BNT162b2 booster dose. 4

Supplementary table 3: Characteristics of individuals who received a BNT162b2 booster dose 5

Supplementary table 4 - Relative VE after booster against SARS-CoV-2 infection compared to individuals with second dose of CoronaVac >180 days. 7

Supplementary table 5 - Relative VE after booster against COVID-19 hospitalizations or death compared to individuals with second dose of CoronaVac >180 days 8

Supplementary Figure 1: Cumulative uptake of vaccination in Brazil. A, B, C: uptake of all vaccines available in Brazil. D, E, F uptake of only CoronaVac. C: (Booster dose) refers to people who have completed vaccine series with any vaccine (CoronaVac, ChAdOx1, Ad26.COV2.S or BNT162b2) and have received a third dose with any vaccine. F: Refers to people who have two doses of CoronaVac and received a booster dose with any vaccine. 9

Supplementary Figure 2: Distribution of variant of concern (VOC) in Brazil from January to November 2021, stratified by region. Data obtained at Fiocruz Genomic Network and/or GISAID-(Visited 2021/12/12). VOI =variant of interest 10

Supplementary figure 3. Percentage by vaccine type in the population with third dose by vaccine in people who have received two doses of CoronaVac in Brazil. 11

Supplementary figure 4- Diagram showing the data linkage process 12

Supplementary table 1. Clinical and sociodemographic characteristics of individuals included in the sensitivity analysis, by SARS-CoV-2 RT-PCR or Antigen test positivity. Vaccination status for CoronaVac is categorized by days since receiving a CoronaVac dose and a booster dose of BNT162b2.

**Characteristic**	Controls, N = 9,486,742[1]	Cases, N = 6,269,322[1]	Overall, N = 15,756,064[1]
**Individuals**	8,395,355 (88%)	5,967,127 (95%)	14,362,482 (91%)
**Type of test**			
**Antigen**	5,113,357 (54%)	2,895,586 (46%)	8,008,943 (51%)
**RT-PCR**	4,373,385 (46%)	3,373,736 (54%)	7,747,121 (49%)
**Age, years**	37 (28, 49)	40 (30, 53)	38 (29, 50)
**Sex-Female**	5,317,888 (56%)	3,253,911 (52%)	8,571,799 (54%)
**Race**			
White	4,364,533 (46%)	2,617,487 (42%)	6,982,020 (44%)
Black	368,831 (3.9%)	236,576 (3.8%)	605,407 (3.8%)
Asian	127,970 (1.3%)	79,872 (1.3%)	207,842 (1.3%)
Mixed	2,576,712 (27%)	1,948,936 (31%)	4,525,648 (29%)
Indigenous	10,428 (0.1%)	5,047 (<0.1%)	15,475 (<0.1%)
(Missing)	2,038,268 (21%)	1,381,404 (22%)	3,419,672 (22%)
**Age group**			
18-59	8,376,735 (88%)	5,308,579 (85%)	13,685,314 (87%)
60-79	967,771 (10%)	829,608 (13%)	1,797,379 (11%)
≥80	142,236 (1.5%)	131,135 (2.1%)	273,371 (1.7%)
**Region of residence**			
Central west	2,294,571 (24%)	1,199,143 (19%)	3,493,714 (22%)
North	418,477 (4.4%)	279,051 (4.5%)	697,528 (4.4%)
Northeast	1,381,162 (15%)	1,146,083 (18%)	2,527,245 (16%)
South	742,602 (7.8%)	645,499 (10%)	1,388,101 (8.8%)
Southeast	4,649,930 (49%)	2,999,546 (48%)	7,649,476 (49%)

**Pregnancy**	71,709 (0.8%)	30,679 (0.5%)	102,388 (0.6%)
**Postpartum period**	4,697 (<0.1%)	3,380 (<0.1%)	8,077 (<0.1%)
**Diabetes Mellitus**	276,714 (2.9%)	305,548 (4.9%)	582,262 (3.7%)
**Obesity**	106,694 (1.1%)	136,156 (2.2%)	242,850 (1.5%)
**Immunosuppression**	61,497 (0.6%)	41,183 (0.7%)	102,680 (0.7%)
**Cardiac Disease**	518,680 (5.5%)	500,168 (8.0%)	1,018,848 (6.5%)
**Chronic Kidney Disease**	30,552 (0.3%)	32,815 (0.5%)	63,367 (0.4%)
**Number of comorbidities**			
0	8,684,596 (92%)	5,501,782 (88%)	14,186,378 (90%)
1	632,592 (6.7%)	554,802 (8.8%)	1,187,394 (7.5%)
≥2	169,554 (1.8%)	212,738 (3.4%)	382,292 (2.4%)
**Previous confirmed infection**	508,897 (5.4%)	60,778 (1.0%)	569,675 (3.6%)
**Hospitalization**	118,669 (1.3%)	650,219 (10%)	768,888 (4.9%)
**Death**	30,448 (0.3%)	187,428 (3.0%)	217,876 (1.4%)
**Hospitalization or Death**	121,373 (1.3%)	659,775 (11%)	781,148 (5.0%)
**Vaccination Status**			
Unvaccinated	5,909,368 (62%)	4,776,983 (76%)	10,686,351 (68%)
CoronaVac	1,244,837 (13.1%)	610,999 (9.7%)	1,855,836 (11.8%)
Other Vaccines	2,335,042 (25%)	881,775 (14%)	3,216,817 (20%)

[1] Median (IQR); n (%)

Supplementary table 2- Vaccine effectiveness against SARS-CoV-2 RT-PCR confirmed symptomatic infection and hospitalization/death using RT-PCR plus antigen detection tests for the diagnosis of COVID-19, by time (days) since second dose of CoronaVac or after BNT162b2 booster dose.

Period post vaccine (days)	Effectiveness against Infection	Effectiveness against severe outcomes
**Second dose**		
0-13	34.4% (33.6-35.2)	65.4% (64.4-66.4)
14-30	51.7% (51.1-52.2)	81.8% (81.2-82.4)
31-60	47.2% (46.7-47.7)	82.1% (81.7-82.5)
61-90	43.2% (42.7-43.8)	79.9% (79.4-80.4)
91-120	41.8% (41.2-42.4)	78.6% (78.0-79.1)
121-150	39.3% (38.6-40.0)	76.8% (76.2-77.5)
151-180	37.0% (36.2-37.9)	75.5% (74.7-76.3)
>180	34.3% (33.2-35.3)	73.3% (72.1-74.5)
**Booster (BNT162b2)**		
0-6	45.9% (42.6-49.0)	83.3% (80.6-85.6)
7-13	84.3% (82.7-85.8)	92.6% (90.8-94.1)
14-30	93.1% (92.2-93.9)	97.3% (96.4-98.0)
>30	89.8% (87.5-91.7)	96.7% (94.8-97.9)

Supplementary table 3: Characteristics of individuals who received a BNT162b2 booster dose

Characteristic	18-59, N = 4,086[1]	60-79, N = 2,339[1]	≥80, N = 1,438[1]	Overall, N = 7,863[1]
**Individuals**	4,080 (99.9%)	2,335 (99.8%)	1,435 (99.8%)	7,850 (99.8%)
**Age, years**	37 (29, 44)	73 (70, 76)	86 (83, 90)	56 (37, 77)
**Sex-Female**	3,265 (79.9%)	1,275 (54.5%)	839 (58.3%)	5,379 (68.4%)
**Race**				
White	2,335 (57.1%)	1,096 (46.9%)	730 (50.8%)	4,161 (52.9%)
Black	177 (4.3%)	51 (2.2%)	32 (2.2%)	260 (3.3%)
Asian	75 (1.8%)	38 (1.6%)	13 (0.9%)	126 (1.6%)
Mixed	913 (22.3%)	458 (19.6%)	283 (19.7%)	1,654 (21.0%)
Indigenous	0 (0.0%)	0 (0.0%)	2 (0.1%)	2 (0.0%)
(Missing)	586 (14.3%)	696 (29.8%)	378 (26.3%)	1,660 (21.1%)
**Region of residence**				
Central-west	847 (20.7%)	422 (18.0%)	432 (30.0%)	1,701 (21.6%)
North	136 (3.3%)	42 (1.8%)	30 (2.1%)	208 (2.6%)
Northeast	583 (14.3%)	247 (10.6%)	168 (11.7%)	998 (12.7%)
South	203 (5.0%)	279 (11.9%)	138 (9.6%)	620 (7.9%)
Southeast	2,317 (56.7%)	1,349 (57.7%)	670 (46.6%)	4,336 (55.1%)
**Pregnancy**	8 (0.2%)	0 (0.0%)	0 (0.0%)	8 (0.1%)
**Post partum period**	0 (0.0%)	0 (0.0%)	0 (0.0%)	0 (0.0%)
**Diabetes Mellitus**	107 (2.6%)	442 (18.9%)	281 (19.5%)	830 (10.6%)
**Obesity**	85 (2.1%)	48 (2.1%)	40 (2.8%)	173 (2.2%)
**Immunossupression**	32 (0.8%)	47 (2.0%)	39 (2.7%)	118 (1.5%)
**Cardiac Disease**	229 (5.6%)	632 (27.0%)	576 (40.1%)	1,437 (18.3%)
**Chronic Kidney Disease**	9 (0.2%)	69 (2.9%)	58 (4.0%)	136 (1.7%)

**Number of comorbidities**				
0	3,702 (90.6%)	1,487 (63.6%)	749 (52.1%)	5,938 (75.5%)
1	315 (7.7%)	535 (22.9%)	437 (30.4%)	1,287 (16.4%)
≥2	69 (1.7%)	317 (13.6%)	252 (17.5%)	638 (8.1%)
**Previous confirmed infection**	736 (18.0%)	127 (5.4%)	67 (4.7%)	930 (11.8%)
**Positive Test (Study Period)**	384 (9.4%)	423 (18.1%)	254 (17.7%)	1,061 (13.5%)
**Hospitalisation**	27 (0.7%)	405 (17.3%)	598 (41.6%)	1,030 (13.1%)
**Death**	2 (0.0%)	72 (3.1%)	139 (9.7%)	213 (2.7%)
**Hospitalisation or Death**	27 (0.7%)	410 (17.5%)	607 (42.2%)	1,044 (13.3%)

1 Median (IQR); n (%);

Supplementary table 4 - Relative VE after booster against SARS-CoV-2 infection compared to individuals with second dose of CoronaVac >180 days.

		Overall	18-59	60-79	≥80
**Only RT-PCR**	CoronaVac >180 days- 2nd dose	Ref			
	Booster (BNT162b2)				
	0-6	7.4 % (-1.7 - 15.7)	9.4% (-3.9-21.0)	1.9% (-14.5-16.0)	1.5% (-24.5-22.1)
	7-13	69.6 % (64.8 - 73.8)	76.6% (69.9-81.8)	63.2% (53.5-70.8)	55.1% (39.0-67.0)
	14-30	88.8 % (86.3 - 90.8)	90.2% (85.9-93.1)	89.9% (85.2-93.1)	80.0% (72.3-85.5)
	>30	73.4 % (64.6 - 80.0)	42.0% (-10.5-69.6)	71.4% (50.5-83.4)	62.6% (44.2-74.9)
**RT-PCR + Rapid antigen test**	CoronaVac >180 days- 2nd dose				
	Booster (BNT162b2)				
	0-6	17.7% (12.6-22.5)	18.3% (10.5-25.4)	17.5% (9.1-25.1)	-1.7% (-19.8-13.7)
	7-13	76.1% (73.7-78.4)	81.4% (77.9-84.4)	75.6% (71.5-79.1)	55.4% (44.8-63.9)
	14-30	89.6% (88.2-90.8)	90.4% (88.1-92.2)	91.4% (89.2-93.1)	80.5% (75.6-84.5)
	>30	84.5% (81.0-87.4)	77.9% (65.3-86.0)	88.6% (82.8-92.5)	74.6% (66.0-81.0)

Supplementary table 5 - Relative VE after booster against COVID-19 hospitalizations or death compared to individuals with second dose of CoronaVac >180 days

		Overall	18-59	60-79	≥80
**Only RT-PCR**	CoronaVac >180 days- 2nd dose	Ref			
	Booster (BNT162b2)				
	0-6	28.9% (13.2-41.7)	56.7% (6.7-79.9)	25.4% (2.8-42.8)	12.8% (-16.6-34.7)
	7-13	68.4% (57.9-76.3)	83.3% (32.1-95.9)	57.2% (38.2-70.3)	62.5% (44.1-74.8)
	14-30	90.1% (85.7-93.1)	91.7% (40.5-98.8)	89.6% (80.6-94.4)	82.0% (72.6-88.2)
	>30	88.3% (78.5-93.7)	99.7% (*)	70.9% (25.6-88.6)	81.8% (63.7-90.9)
**RT-PCR + Rapid antigen test**	CoronaVac >180 days- 2nd dose	Ref			
	Booster (BNT162b2)				
	0-6	37.4% (27.0-46.3)	70.4% (40.0-85.4)	42.4% (29.2-53.1)	15.6% (-6.3-32.9)
	7-13	72.4% (65.5-77.9)	90.2% (60.6-97.6)	72.5% (62.6-79.8)	62.9% (49.5-72.8)
	14-30	89.8% (86.4-92.3)	91.6% (66.1-97.9)	92.4% (87.4-95.4)	83.1% (76.3-88.0)
	>30	87.7% (80.5-92.3)	43.9% (-306.7-92.3)	87.7% (69.7-95.0)	82.4% (70.5-89.5)

Supplementary Figure 1: Cumulative uptake of vaccination in Brazil. A, B, C: uptake of all vaccines available in Brazil. D, E, F uptake of only CoronaVac. C: (Booster dose) refers to people who have completed vaccine series with any vaccine (CoronaVac, ChAdOx1, Ad26.COV2.S or BNT162b2) and have received a third dose with any vaccine. F: Refers to people who have two doses of CoronaVac and received a booster dose with any vaccine.

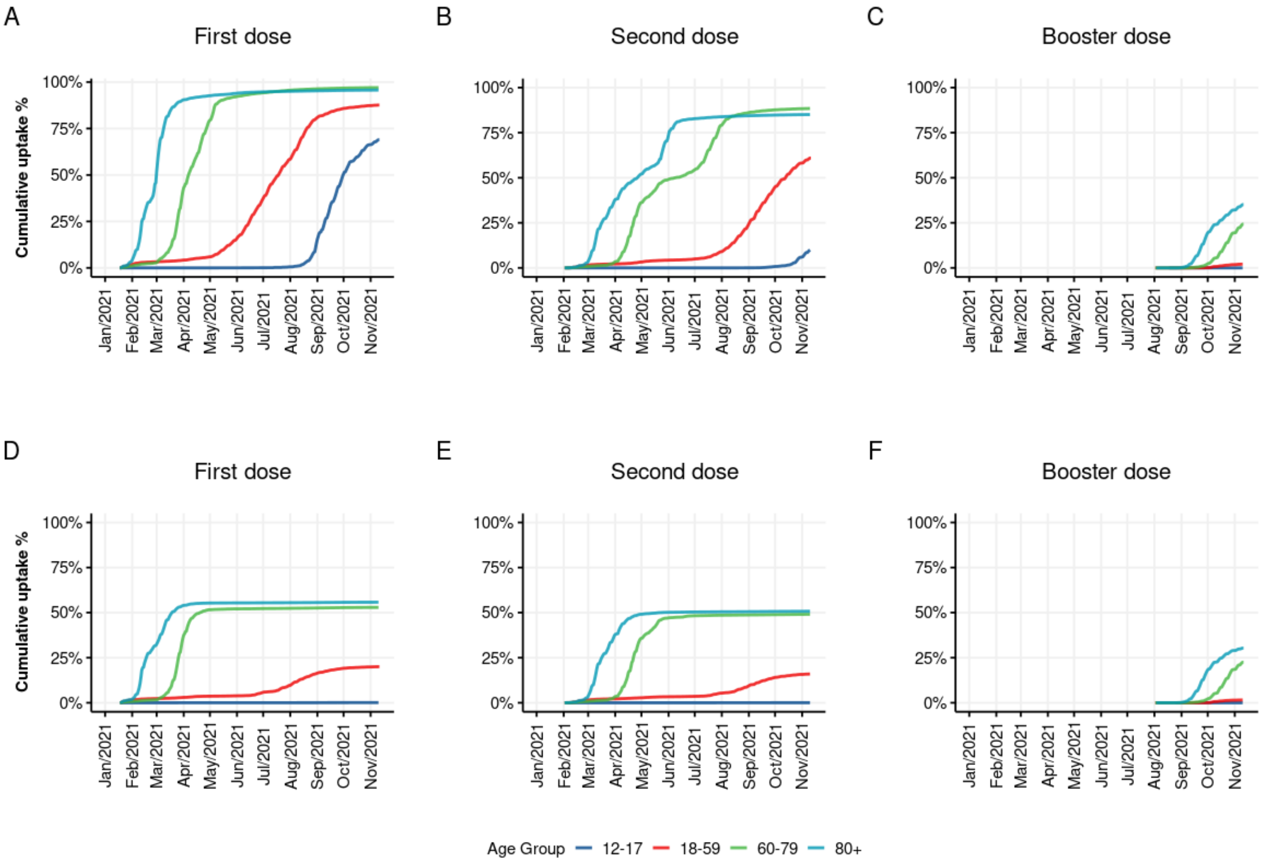

Supplementary Figure 2: Distribution of variant of concern (VOC) in Brazil from January to November 2021, stratified by region. Data obtained at Fiocruz Genomic Network and/or GISAID- (Visited 2021/12/12). VOI =variant of interest

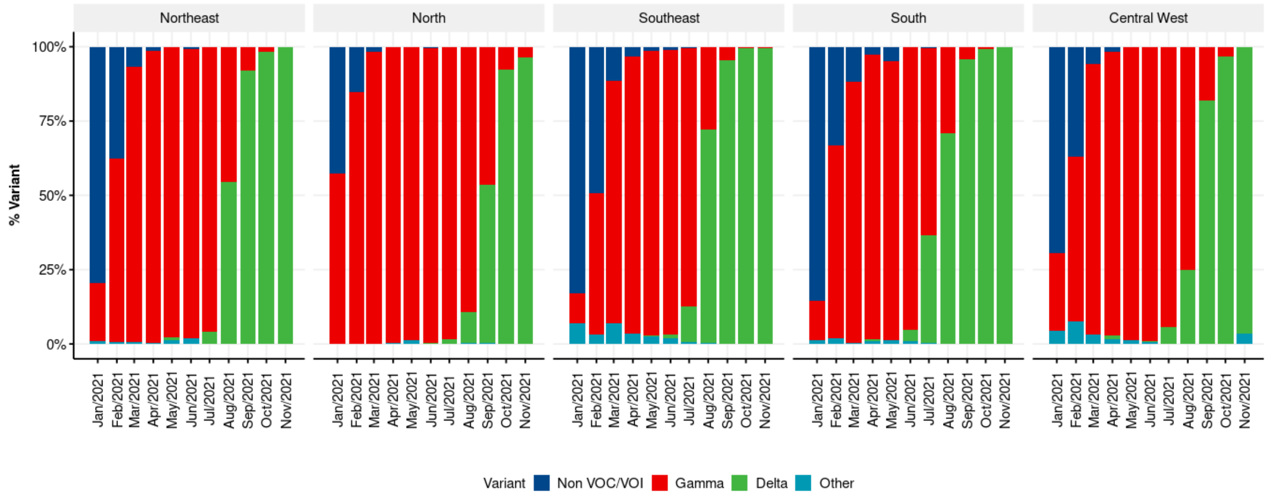

Supplementary figure 3. Percentage by vaccine type in the population with third dose by vaccine in people who have received two doses of CoronaVac in Brazil.

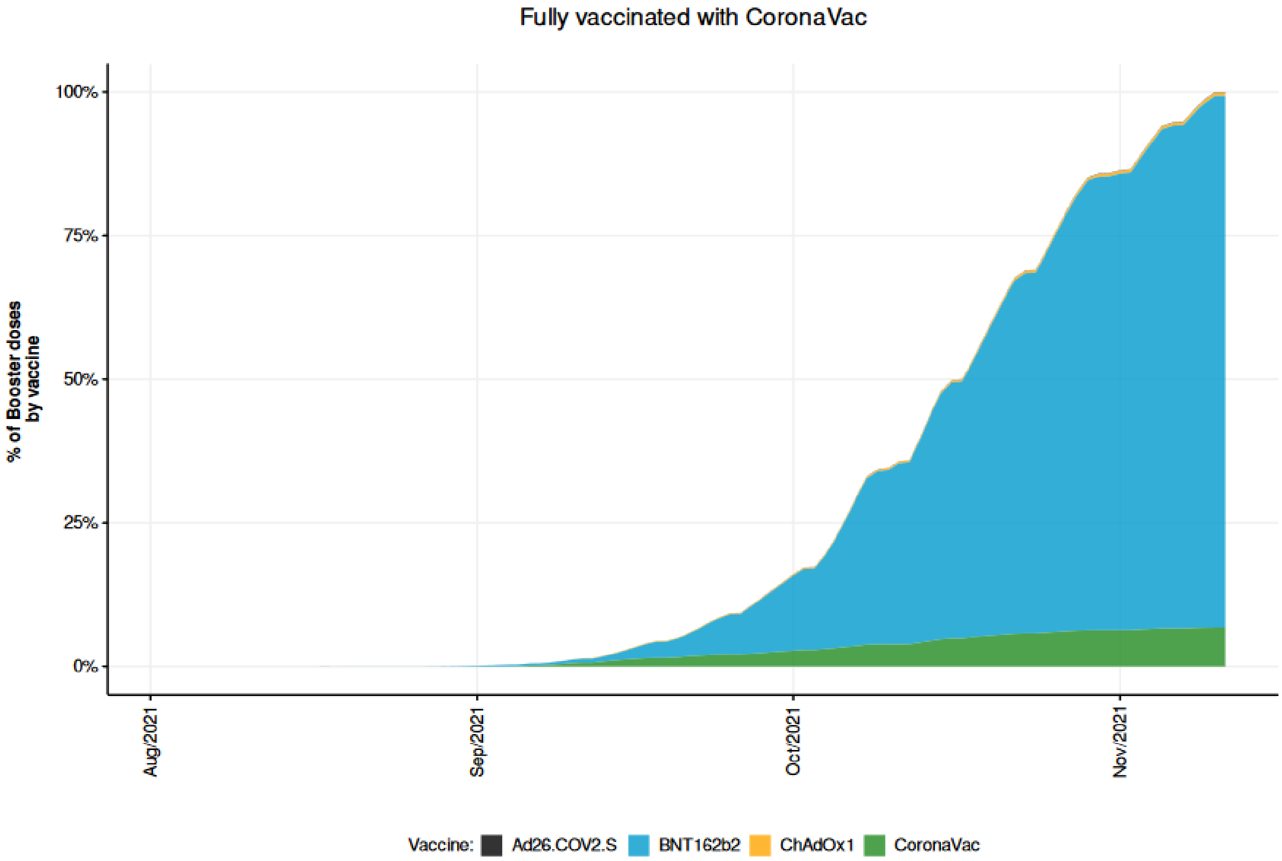

Supplementary figure 4- Diagram showing the data linkage process

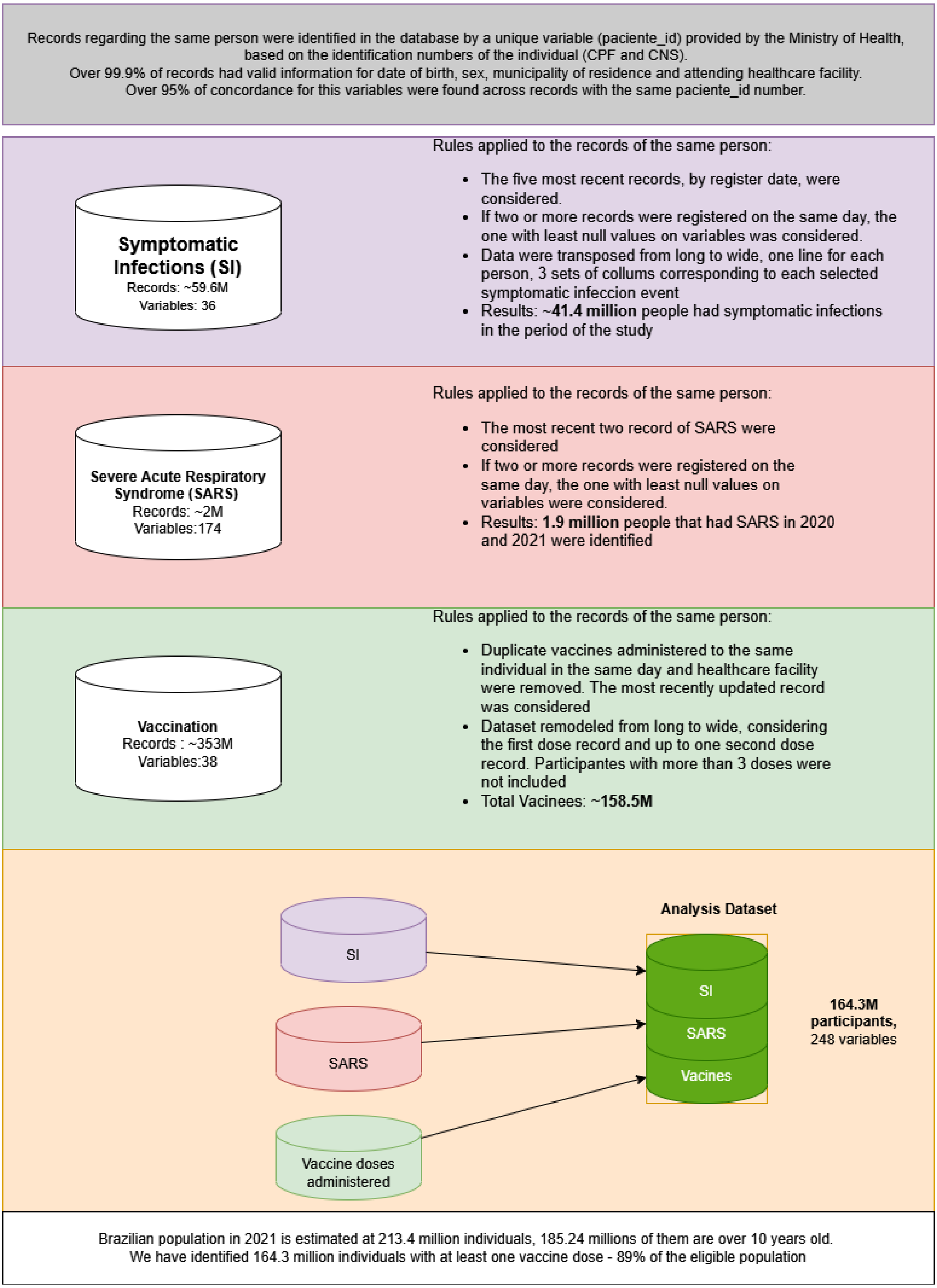

Supplement: Supplementary file 1 — Supplementary Tables 1–5 and Figs. 1–4. [file 41591_2022_1701_MOESM1_ESM.pdf]
