# Supplementary material for: Vaccine effectiveness of heterologous CoronaVac plus BNT162b2 in Brazil
Source: Nat Med. 2022 Feb 9;28(4):838–43. doi: 10.1038/s41591-022-01701-w (PMC9018414; doi:10.1038/s41591-022-01701-w)
Supplement: Supplementary file 2 — Reporting Summary [file 41591_2022_1701_MOESM2_ESM.pdf]

Corresponding author(s): *Double-blind peer review submissions: write DBPR and your manuscript number here instead of author names.*

Last updated by author(s): *YYYY-MM-DD*

Reporting Summary

Nature Portfolio wishes to improve the reproducibility of the work that we publish. This form provides structure for consistency and transparency in reporting. For further information on Nature Portfolio policies, see our Editorial Policies and the Editorial Policy Checklist.

Statistics

For all statistical analyses, confirm that the following items are present in the figure legend, table legend, main text, or Methods section.

n/a	Confirmed	
☐	☒	The exact sample size (*n*) for each experimental group/condition, given as a discrete number and unit of measurement
☐	☒	A statement on whether measurements were taken from distinct samples or whether the same sample was measured repeatedly
☒	☐	The statistical test(s) used AND whether they are one- or two-sided<br>*Only common tests should be described solely by name; describe more complex techniques in the Methods section.*
☐	☒	A description of all covariates tested
☐	☒	A description of any assumptions or corrections, such as tests of normality and adjustment for multiple comparisons
☐	☒	A full description of the statistical parameters including central tendency (e.g. means) or other basic estimates (e.g. regression coefficient) AND variation (e.g. standard deviation) or associated estimates of uncertainty (e.g. confidence intervals)
☒	☐	For null hypothesis testing, the test statistic (e.g. *F*, *t*, *r*) with confidence intervals, effect sizes, degrees of freedom and *P* value noted<br>*Give P values as exact values whenever suitable.*
☒	☐	For Bayesian analysis, information on the choice of priors and Markov chain Monte Carlo settings
☒	☐	For hierarchical and complex designs, identification of the appropriate level for tests and full reporting of outcomes
☒	☐	Estimates of effect sizes (e.g. Cohen's *d*, Pearson's *r*), indicating how they were calculated

Our web collection on statistics for biologists contains articles on many of the points above.

Software and code

Policy information about availability of computer code

Data collection	R version 4.1.1
Data analysis	R version 4.1.1; packages: mgcv version 1.8-36; tidyverse version 1.3.1

For manuscripts utilizing custom algorithms or software that are central to the research but not yet described in published literature, software must be made available to editors and reviewers. We strongly encourage code deposition in a community repository (e.g. GitHub). See the Nature Portfolio guidelines for submitting code & software for further information.

Data

Policy information about availability of data

All manuscripts must include a data availability statement. This statement should provide the following information, where applicable:

- Accession codes, unique identifiers, or web links for publicly available datasets
- A description of any restrictions on data availability
- For clinical datasets or third party data, please ensure that the statement adheres to our policy

One of the study coordinators (MB-N) signed a term of responsibility on using each database made available by the Ministry of Health (MoH). Each member of the research team signed a term of confidentiality before accessing the data. Data was manipulated in a secure computing environment, ensuring protection against data leakage. The Brazilian National Commission in Research Ethics approved the research protocol (CONEP approval number 4.921.308). Our agreement with MoH for accessing the databases patently denies authorization of access to a third party. Any information for assessing the databases must be addressed to the Brazilian MoH at https://datasus.saude.gov.br/, and requests can be addressed to datasus@saude.gov.br. Herein we used anonymized secondary data following the Brazilian Personal Data Protection General Law (LGPD), but it is vulnerable to re-identification by third parties, as they contain dates of relevant health events regarding the

same person. To protect the research participants' privacy, the approved Research Protocol (CONEP approval number 4.921.308) authorizes only the dissemination of aggregated data, such as the data presented here.

Field-specific reporting

Please select the one below that is the best fit for your research. If you are not sure, read the appropriate sections before making your selection.

☒ Life sciences ☐ Behavioural & social sciences ☐ Ecological, evolutionary & environmental sciences

For a reference copy of the document with all sections, see nature.com/documents/nr-reporting-summary-flat.pdf

Life sciences study design

All studies must disclose on these points even when the disclosure is negative.

Sample size

Given that the sample sizes were based on national linked datasets with our sample size only excluding individuals that do not fit the eligibility criteria, the sample size for each sub-study can be considered sufficient. Of 7,314,318 individuals tested between January 18,2021 and November 11, 2021.

Data exclusions

We excluded: (i) individuals younger than 18 years; (ii) individuals who received a different vaccine for the second dose from the first; (iii) individuals whose time interval between the first and second doses was less than 14 days; (iv) tests with missing information of age, sex, city of residence or sample collection date; (v) negative test within 14 days of a previous negative test; (vi) negative test followed by a positive test up to 7 days; (vii) any test after a positive test up to 90 days; (viii) tests with a symptom onset date greater than notification date

Replication

For replication, additional analyses were conducted to estimate vaccine effectiveness: using rapid antigen test in addition to RT-PCR test as diagnostic criteria and using the using as comparison group individuals with more than 180 days post second dose of CoronaVac. All analyses confirmed/reproduced estimates of vaccine effectiveness obtained in the main analysis.

Randomization

Not applicable (this is an observational study)

Blinding

Not applicable (this is an observational study)

Reporting for specific materials, systems and methods

We require information from authors about some types of materials, experimental systems and methods used in many studies. Here, indicate whether each material, system or method listed is relevant to your study. If you are not sure if a list item applies to your research, read the appropriate section before selecting a response.

Materials & experimental systems

n/a	Involved in the study
☒	☐ Antibodies
☒	☐ Eukaryotic cell lines
☒	☐ Palaeontology and archaeology
☒	☐ Animals and other organisms
☒	☐ Human research participants
☒	☐ Clinical data
☒	☐ Dual use research of concern

Methods

n/a	Involved in the study
☒	☐ ChIP-seq
☒	☐ Flow cytometry
☒	☐ MRI-based neuroimaging
